# EpCAM based capture detects and recovers circulating tumor cells from all subtypes of breast cancer except claudin-low

**DOI:** 10.18632/oncotarget.5977

**Published:** 2015-10-19

**Authors:** Alexander Ring, Neal Mineyev, Weizhu Zhu, Emily Park, Chip Lomas, Vasu Punj, Min Yu, Dany Barrak, Victoria Forte, Tania Porras, Debu Tripathy, Julie E. Lang

**Affiliations:** ^1^ Department of Surgery, Keck School of Medicine, University of Southern California, Los Angeles, CA 90033, USA; ^2^ Norris Comprehensive Cancer Center, University of Southern California, Los Angeles, CA 90033, USA; ^3^ Department of Surgery, Lenox Hospital New York, New York, NY 10065, USA; ^4^ Advanced Cell Diagnostics, Research and Development, Hayward, CA 94545, USA; ^5^ BD Biosciences, Research and Development, San Jose, CA 95131, USA; ^6^ Division of Hematology, Keck School of Medicine, University of Southern California, Los Angeles, CA 90033, USA; ^7^ Eli and Edythe Broad Center for Regenerative Medicine and Stem Cell Research at USC, Keck School of Medicine, University of Southern California, Los Angeles, CA 90033, USA; ^8^ Department of Breast Medical Oncology, The University of Texas MD Anderson Cancer Center, Houston, TX 77030, USA

**Keywords:** breast cancer, circulating tumor cells, IE/FACS, EpCAM

## Abstract

**Purpose:**

The potential utility of circulating tumor cells (CTCs) as liquid biopsies is of great interest. We hypothesized that CTC capture using EpCAM based gating is feasible for most breast cancer subtypes.

**Results:**

Cancer cells could be recovered from all intrinsic subtypes of breast cancer with IE/FACS, however, claudin-low cell lines showed very low capture rates compared to the four other groups (*p* = 0.03). IE/FACS detection of CTC mimic cells was time sensitive, emphasizing controlling for pre-analytic variables in CTC studies. Median fluorescent intensity for flow cytometry and RNA flow cell type characterization were highly correlated, predicting for CTC isolation across molecular subtypes. RNA-Seq of IE/FACS sorted single cell equivalents showed high correlation compared to bulk cell lines, and distinct gene expression signatures compared to PB.

**Materials and Methods:**

Ten cell lines representing all major subtypes of breast cancer were spiked (as CTC mimics) into and recovered from peripheral blood (PB) using immunomagnetic enrichment followed by fluorescence-activated cell sorting (IE/FACS). Flow cytometry and RNA flow were used to quantify the expression of multiple breast cancer related markers of interest. Two different RNA-Seq technologies were used to analyze global gene expression of recovered sorted cells compared to bulk cell lines and PB.

**Conclusions:**

EpCAM based IE/FACS detected and captured a portion of spiked cells from each of the 10 cell lines representing all breast cancer subtypes, including basal-like but not claudin-low cancers. The assay allows for the isolation of high quality RNA suitable for accurate RNA-Seq of heterogeneous rare cell populations.

## INTRODUCTION

Metastasis is responsible for the vast majority of breast cancer related deaths [1]. The shedding of tumor cells from their primary site into the systemic circulation via hematogenous spread is thought to be a major cause of distant metastasis [2–4]. These circulating tumor cells (CTCs) are extremely rare, with approximately one cancer cells per 10^6−7^ white blood cells (WBC), which makes their detection and capture formidably challenging [5]. CTCs have been demonstrated to be prognostic in all stages of breast cancer [6–9]. Several methods exist for enumerating CTCs, including filtration and affinity based strategies [6, 10, 11]. Filtration based methods rely mostly on marginal size difference between CTCs and WBCs, with 13 μm and 10 μm diameter on average, respectively [12]. Affinity based CTC assays most commonly involve the epithelial cell surface marker Epithelial Cell Adhesion Molecule (EpCAM), a transmembrane glycoprotein. Breast cancer may be classified into intrinsic subtypes based on primary tumor transcriptional profiling that describe the heterogeneity of disease [13, 14]. Intrinsic subtypes predict for metastasis patterns and risk of recurrence in breast cancer [13, 15]. Sieuwerts *et al* reported that the U.S. Food and Drug Administration-approved CellSearch Assay (Janssen Diagnostics, Raritan NJ) was unable to detect CTCs of the normal-like intrinsic subtype [16]. Recent studies have questioned the existence of the normal like subtype, and raised concerns about it being a potential artifact of normal breast tissue contamination and low sample cancer cellularity [13]. Instead, a claudin-low intrinsic subtype of breast cancer has been described as a subset of basal-like breast cancers characterized by low to absent expression of claudin 3 and E-cadherin (CDH1), as well as stem-cell like features [17, 18]. In this report, we implement a newly described technique of immunomagnetic enrichment followed by fluorescence-activated cell sorting (IE/FACS) for the isolation of spiked cancer cells (CTC mimics) from blood suitable for use for whole transcriptome analysis at the single cell level [19, 20]. Unlike other methods, which usually have substantial inherent leukocyte contamination, our workflow for spiked cell isolation enables us to efficiently enrich and extract these cells with high purity. The aim of this paper was to evaluate the ability of multi-marker IE/FACS based on immunomagnetic separation with EpCAM to recover spiked cancer cells across the spectrum of intrinsic subtypes in breast cancer. We hypothesized that CTC capture using EpCAM based gating is feasible for most breast cancer subtypes. A secondary aim of this paper was to report the accuracy of next generation sequencing (NGS) of IE/FACS sorted spiked cells.

## RESULTS

### Recovery rates

**Figure 1:**
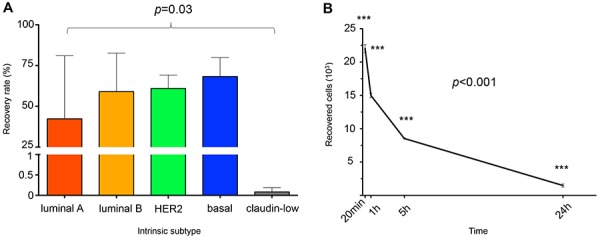
**A.** Bar graph representation of recovery rates (*n* = 3 for each cell line). Overall recovery rates for PBS 51.4%, PB 39.5%. Recovery rates are statistically significantly different based on subtype (*p* = 0.02). **B.** Time course experiment demonstrating the rapid decline in cell recovery as a function of blood draw-to-isolation time (*n* = 3 per time point).

Table 1 provides the IE/FACS recovery rates from phosphate buffered saline (PBS) and peripheral blood (PB) for all 10 cell lines and according to molecular subtype [20]. The overall mean recovery rates were 51.4% from PBS and 39.5% from PB. The specific cell type being analyzed was a more significant source of variation (*p* = 0.03) than was whether measurements were made from PBS or PB (*p* = 0.26). Figure 1A demonstrates that the 2 claudin-low cell lines had lower IE/FACS recovery rates than the other 4 intrinsic subtypes (*p* = 0.03). A time course experiment revealed that the time from blood draw to cell harvest is critical for the maximization of viable cell retrieval (Figure 1B). Within one hour, a reduction of 32% was observed in CTC mimic cells enumerated via IE/FACS from blood specimens drawn into EDTA tubes.

**Table 1:** IE/FACS recovery rates

Cell line	PBS (% recovered)	PB (% recovered)	ER	PR	HER2	Subtype [20]
T47D	28.9	12.6	+	+	−	Luminal A
MCF7	99.3	28.6	+	+/−	−	Luminal A
BT474	94.1	42.6	+	+/−	+	Luminal B
ZR-75-1	49.4	50	+	+/−	+	Luminal B
SKBr3	68.0	49.5	−	−	+	Her2
MDA-MB-453	60.7	65.7	−	−	+	Her2
SUM149	64.9	69.5	−	−	−	Basal
SUM190	83.3	55.4	−	−	+	Basal
MDA-MB-231	0.1	0.23	−	−	−	Claudin-low
Hs578T	0.002	0.004	−	−	−	Claudin-low

### Purity of the sorted cells

**Figure 2:**
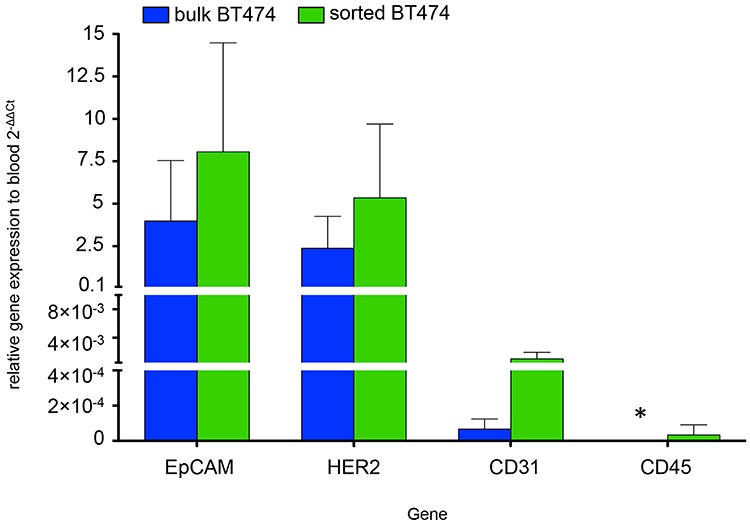
qRT-PCR comparing gene expression of bulk BT474 (BT474b) (blue) and sorted BT474 (BT474s) (green) to PB Results are represented as fold change (2^−ΔΔCt^) (*n* = 3). Epithelial cell specific genes not expressed by blood cells were significantly higher expressed in bulk and sorted BT474 compared to blood: EpCAM (4-fold BT474b, 8.1-fold BT474s), HER2 (2.4-fold BT474b, 5.3-fold BT474s). Expression of genes predominantly found on blood and endothelial cells was significantly lower in bulk and sorted BT474 compared to PB: CD45 (not detected in BT474b (*), 0.00003-fold BT474s), CD31 (0.0007-fold BT474b, 0.001 BT474s). Gene expression differences between sorted and bulk BT474 was not statistically significant (2-way ANOVA, *p* = 0.2).

To verify cancer cell purity after recovery from blood, BT-474 cells were spiked into PB and sorted using our IE/FACS assay. TaqMan real-time reverse transcription polymerase chain reaction (qRT-PCR) comparison of PB markers (CD45 and CD31) showed similarly low expression levels in BT474 bulk and sorted cells as well as a significantly higher expression in blood (Figure 2). Markers highly expressed on normal and cancerous epithelial breast cells (EpCAM and HER2) highly correlated between BT474 bulk and sorted cells, with significantly higher expression levels compared to PB (Figure 2). In summary, this data indicated high purity of sorted cells using IE/FACS, with minimal blood cell contamination.

### Cell characterization using RNA flow and FACS

**Figure 3:**
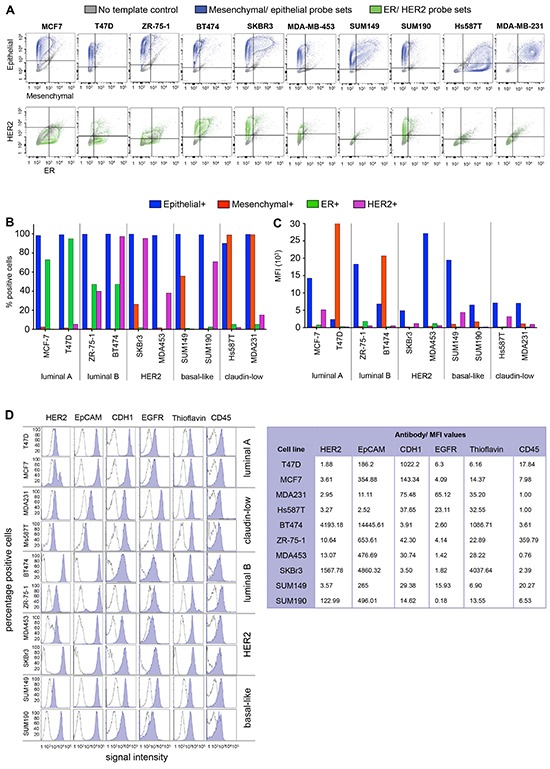
Characterization of breast cancer subtypes **A–C.** RNA flow. **A.** Density plots representing mesenchymal (FITC, x-axis) and epithelial (PE-YG, y-axis) marker RNA expression (top row), as well as ER (APC, x-axis) and HER2 (BV421, y-axis) RNA expression in 10 breast cancer cell lines. **B.** The number of positive cells (panel A) correlated with subtype classification for all probe sets, except for epithelial markers. **C.** MFI revealed bright subpopulations within each cell line, independent of subtype. Cells expressing high levels of mesenchymal markers were present in luminal subtypes (T47D, BT474), while the basal like cell line SUM149 showed high expression of epithelial markers. **D.** FACS. Epithelial cell (EpCAM, CDH1) and breast cancer marker-expression (HER2, EGFR) correlated with the clinical subtype classification. Staining with the benzothiazole thioflavin (TF) successfully captured nucleated cells. Expression of the white blood cell marker CD45 was equally low in all cell lines.

Density plots demonstrate the cell line specific distribution of all four probe sets (ER, HER2, mesenchymal and epithelial) used for RNA flow (Figure 3A). The majority of cells in all 10 cell lines expressed epithelial markers present in the multi-marker probes (between 90 and 99.7%) (Figure 3B). The median fluorescence intensity (MFI) for epithelial markers was not statistically significantly different based on intrinsic subtype for RNA flow cytometry (*p* = 0.45) although MFIs for individual cell line types were highly variable (range 2376–27,148) (Figure 3C). Similarly, mesenchymal marker MFI was also not statistically significantly different based on intrinsic subtype (*p* = 0.4), while considerable variations between cell lines types were noted (range 200–33,679) (Figure 3B). The percentage of positive cells differed depending on cell line and subtype for mesenchymal markers (Figure 3B). The claudin-low cell lines MDA-MB-231 and Hs578T contained 99.1% and 98.8% positive cells, respectively. The basal-like cell lines SUM149 and SUM190 exhibited 55.8% and 0.8% positive cells, respectively. Both luminal (A and B) subtypes displayed low expression levels of the mesenchymal markers (Luminal A: T47D 1.5%, MCF7 2.5%; Luminal B: BT474 0.6%, ZR-75-1 1.2%). RNA flow results for ER (FACS positive cells lines MFI values range 216–1799, FACS negative cell lines MFI 129–317) and HER2 (MFI range for HER2 positive = 612–5120; MFI range for HER2 negative = 185–927) are presented in Figure 3C. RNA expression showed a wide dynamic range depending on the cell line. Figure 3D provides histograms to display single antibody staining fluorescence results for 6 breast cancer cell markers (HER2, EpCAM, CDH1, EGFR, Thioflavin, CD45) for the purpose of characterizing cell line heterogeneity. EpCAM MFI were greater than 186 for most cell lines with the exception of the claudin low cell lines (Hs578T and MDA-MB-231), which both showed virtually no separation between stained and unstained controls for the EpCAM antibody (MFI range 2.52–11.11 for claudin-low versus 186.2–14,445.6 for all others). Subtype specific analysis showed statistically significant MFI variation (*p* = 0.04).

### Helicos low abundant RNA sequencing

**Figure 4:**
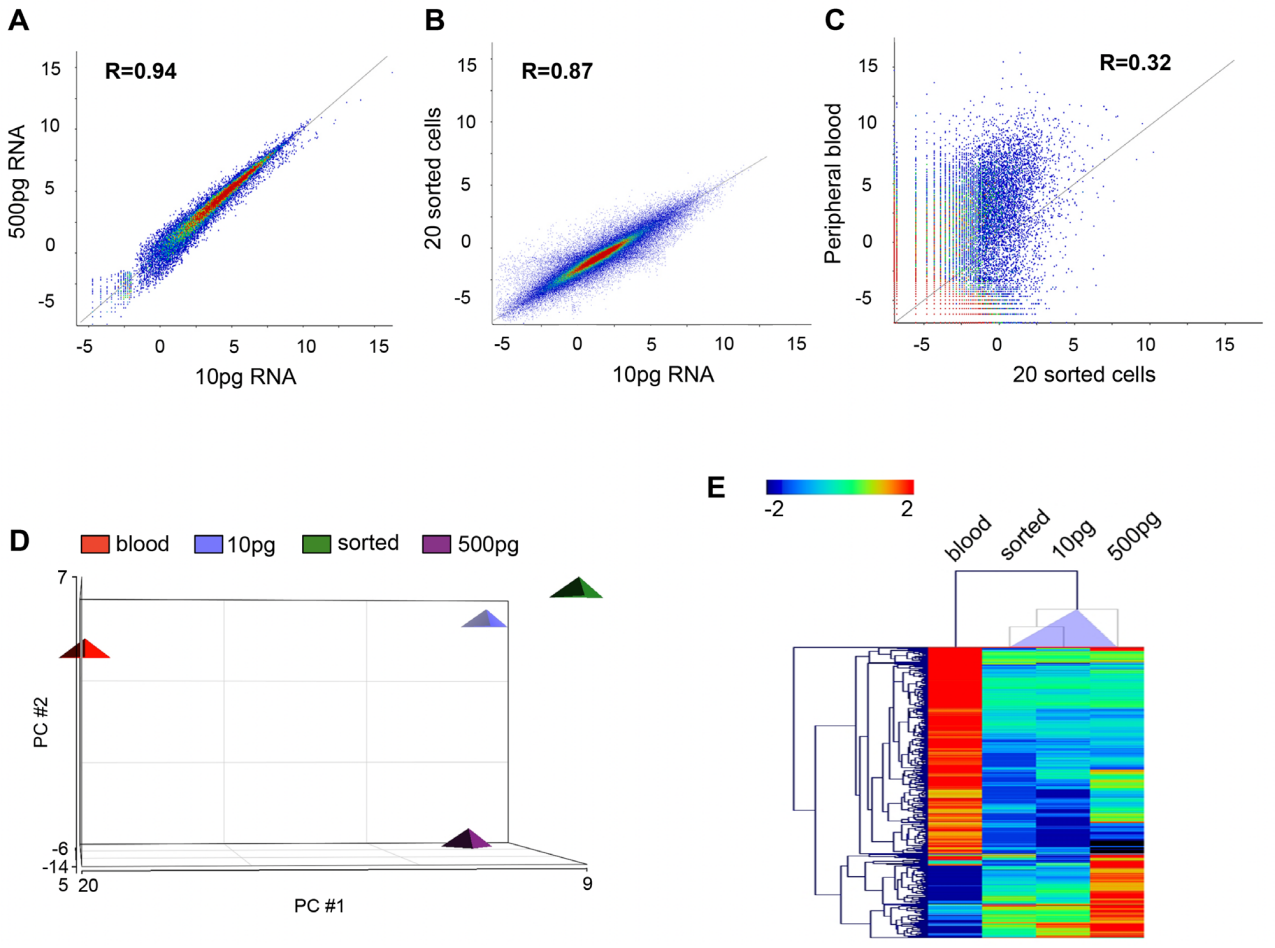
Helicos sequencing data **A–C.** Scatter plots demonstrating high correlation between low (10 pg) and high (500 pg) RNA inputs (*R* = 0.94, **A.**) low RNA input and sorted cells (*R* = 0.87, **B.**) but no correlation between sorted cells and PB (*R* = 0.32, **C.**) (Pearson's correlation analysis). **D.** PCA analysis demonstrated similarity between sorted BT474 and different RNA inputs from bulk BT474, and separation from PB. **E.** HC of 274 genes, which show differential expression of FC ≥ ± 2 (FDR adjusted *p* < 0.05) and differentiate BT474 (sorted cells and bulk cell RNA) from PB blood.

**Figure 5:**
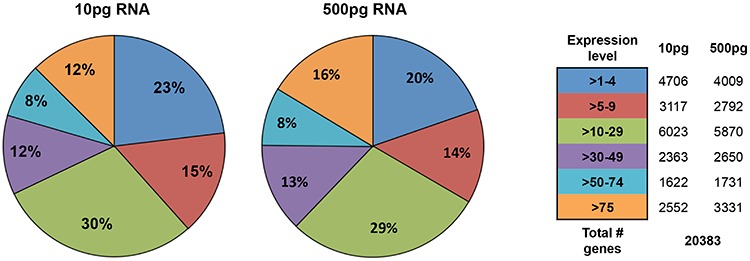
Helicos sequencing gene expression detection sensitivity A detailed analysis of detected genes based on transcript abundance shows high correlation between low and high RNA inputs.

To verify if RNA-Seq is feasible for low RNA inputs, 10 pg and 500 pg of total RNA were first analyzed using Helicos Low Abundant Molecule Next Generation Sequencing a significant correlation was observed for 10 and 500 pg RNA inputs (*R* = 0.94, Figure 4A). RNA derived from twenty sorted BT474 cells spiked into PB highly correlated to 10 pg total RNA from bulk BT474 cells and validated that the IE/FACS CTC mimic cell isolation strategy did not alter gene expression (correlation co-efficient *R* = 0.87, Figure 4B). A comparison of sorted BT474 cells with RNA obtained from PB demonstrated statistically significant separation of spiked cancer cells from PB (*R* = 0.32, Figure 4C). A principal component analysis comparing sorted BT474 cells and BT474 bulk cell RNA inputs shows similarity between these samples, with a clear separation from PB (Figure 4D). Unsupervised hierarchical clustering (HC) for these samples identified 274 differentially expressed genes (FC ≥ ± 2, FDR adjusted *p* < 0.05) that are shared between all BT474 samples compared to PB (Figure 4E). Further analysis of a wide dynamic range of gene expression demonstrated that low abundance gene transcripts (absolute expression 1–4) as well as highly abundant transcripts (absolute expression > 50) could be detected at similar levels, independent of RNA input (Figure 5).

### Illumina hi-seq

**Figure 6:**
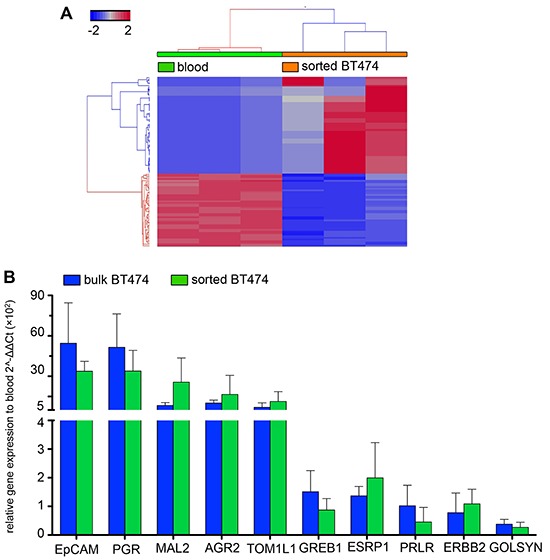
Illumina high seq. data **A.** Unsupervised hierarchical clustering of RNA-Seq data from PB and sorted BT474. A signature of 123 differentially expressed genes (FC ≥ ± 2, FDR adjusted *p* < 0.05) clearly separate sorted cancer cells from PB (*n* = 3 each). **B.** qRT-PCR validation. Ten genes selected from the RNA-Seq results show similar expression trends in BT474 sorted and bulk compared to PB. Expression levels are not statistically significantly different between sorted and bulk cells (2-way ANOVA, *p* = 0.7).

Whole transcriptome analysis was performed on 20 and 100 IE/FACS-sorted cell aliquots and compared to PB, which did not show any statistically significant correlation (*R* = 0.5, PB vs. 20 cells; *R* = 0.52, PB vs. 100 cells) (Supplementary Figure S1). Comparing different numbers of sorted cells to both bulk RNA and to each other showed statistically significant correlation (20 cells vs. bulk. *R* = 0.88; 100 cells vs. bulk, *R* = 0.91; 20 cells vs. 100 cells, *R* = 0.92) (Supplementary Figure S1). Differential gene express analysis comparing PB and sorted cells identified a total of 123 genes (FC ≥ ± 2, FDR adjusted *p* < 0.05) differentiating isolated spiked cancer cells from blood RNA (Figure 6A). qRT-PCR validation of the RNA-Seq results for the expression of 10 genes selected from the RNA-Seq. gene list (GREB1, PRLR, AGR2, ESRP1, GOLSYN, MAL2, PGR, KRT8, ERBB2 and TOM1L1) confirmed the results and showed similar expression levels in sorted BT474 and BT474 bulk RNA, but significantly different expression compared to PB (Figure 6B).

## DISCUSSION

In the current study we sought to establish whether an EpCAM based IE/FACS CTC assay can capture the full spectrum of breast cancer subtypes. Furthermore, we validated whether this approach allows for the isolation and whole transcriptome analysis of CTC gene expression. Both research questions have direct impactions for breast cancer diagnosis and treatment. Metastatic breast cancers (MBC) have been documented to change their clinical biomarker profiles when compared to a primary tumor [21, 22], with direct implication for targeted therapeutic approaches. Several authors have reported discordance of up to 19% for ER between primary tumors and metastatic sites, without prior treatment [23, 24]. Niikura *et al*. showed that 24% of patients with HER2 positive primary tumors had HER2 negative metastases [25]. Meng *et al*. demonstrated that CTCs as liquid biopsies might be superior to capture the dynamic changes between primary tumor and distant metastasis [26]. They also demonstrated HER2 amplification in CTCs of patients with recurrent HER2 negative disease. Most remarkably, treatment with trastuzumab based on this information resulted in beneficial outcomes for a subset of patients. Thus, the true biology of MBC and current options for targeted therapies cannot be ascertained by profiling what the tumor once was and CTCs could be a valuable tool to inform therapeutic decisions. We have previously established a method for the isolation and expression profiling of EpCAM positive CTCs [27], which allows for the rapid and efficient isolation of viable cells and high quality RNA for downstream analysis. In the current study, using 10 breast cancer cell lines representing the heterogeneity of breast cancer, we were able to demonstrate that using IE/FACS, all cell lines were extracted with high efficiency from PBS and PB spikes, except for the claudin-low subtype cell lines. Negative control PB specimens from healthy individuals could successfully define a consistent gating strategy that captured zero cells from healthy individuals and permitted acquiring a portion of CTC mimic cells from each of the 10 cell lines. Significant variation occurred in recovery rates of spiked and sorted cells depending on cell line and subtype, with the lowest recoveries from the claudin-low subtype. Several studies have shown that CTCs can exhibit substantial pleomorphism, including stem like features [28], and could (co-) express epithelial, mesenchymal, and cancer stem cell (CSC) markers. Future studies could include antibodies against mesenchymal/EMT as well CSC markers to avoid missing clinically relevant CTC populations [29].

We used FACS and RNA flow to characterize all 10 cell lines based on widely used breast cancer markers. FACS reliably separated the cell lines according to intrinsic subtype. RNA flow overall confirmed subtype differences in epithelial and mesenchymal transcript expression. Previous studies demonstrated that the triple negative claudin-low subtype expressed low levels of EpCAM. RNA flow results supported the low abundance (low MFI) of EpCAM transcripts in the claudin-low subtype cells. Nevertheless, EpCAM transcripts were detected in 100% of the cells in the claudin-low subtype, albeit at very low levels (MFI at least 16-fold less compared to other subtypes). Surprisingly, lower MFI were also detected for T47D, while EpCAM antibody staining was highly positive. This may be driven by the unique expression pattern of T47D, which showed high mesenchymal marker MFI. Overall, RNA flow for mesenchymal markers showed higher expression levels in basal like cell lines (Hs578T, MDA-MB-231, SUM149) than the remaining cell lines [16]. Nevertheless, subpopulations with high expression levels of mesenchymal markers were detected in luminal subtype cell lines (T47D, BT474). Overall, RNA flow cytometry results did not fully correlate with known protein expression levels for the 10 cell lines - with considerable overlap between ER positive lines and ER negative lines. HER2 gene expression by RNA flow cytometry correctly identified 100% of the 5 HER2 positive cell lines; however, overlapping intervals were found with 20% (1/5) HER2 negative cell lines (T47D) showing MFI in the HER2 positive range. The discrepancies between FACS and RNA flow should be further investigated. This could be dependent on the specific cell type and could identify mechanisms by which cancer cells change their phenotype (e.g. transcriptional vs. translational alterations). It is also possible that these results are an indication that most cell lines tested are of epithelial origin (EpCAM expression), with some undergoing an EMT like process (mesenchymal marker expression), which has been linked to CSC like features [30, 31].

Validation of cell purity using qRT-PCR showed good concordance between bulk and IE/FACS sorted cells. However, the true capability of our EpCAM-based IE/FACS strategy to isolate rare cells from heterogeneous patient CTC samples with high purity will have to be investigated in subsequent studies. Our results demonstrated that the time interval between blood draw and processing of IE/FACS sorted cells drastically impacted the rate of viable cell detection, emphasizing the need for an expeditious and standardized sample processing practice. Some previous studies paid little attention to this aspect, with greatly varying sample processing times of up to 72 h [16]. Utilizing our rapid processing algorithm should minimize pre-analytic variability such as cell loss and RNA degradation. Using two independent NGS methods, we demonstrated that, independent of cell number or amount of input RNA, our assay could capture rare cell cancer cell populations, preserve high quality RNA and achieve accurate NGS from single or small pools of cells. Using sorted cells and bulk cells or their respective isolated RNA showed that the EpCAM based IE/FACS sorting and capture does not seem to affect the transcriptome of cancer cells at a significant level, as high correlation in gene expression between both populations was found. Presently, relatively few clinically actionable predictive biomarkers exist for breast cancer [32]. Our unbiased whole transcriptome analysis of CTCs could potentially be used to identify novel biomarkers as well as actionable drug targets.

## MATERIALS AND METHODS

### Cell lines and culture conditions

Ten breast cancer cell lines were acquired from the ATCC and authenticated by short tandem repeat profiling using the Identifiler polymerase chain reaction (PCR) kit (AmpFSTR, Applied Biosystems, Foster City, CA) at the University of Arizona Genetics Core. Cell lines were stratified according to subtype [33]: HER2 positive (SKBR3, MDA-MB-453), luminal A (T47D, MCF7), luminal B (BT474, ZR-75-1), basal-like (SUM149, SUM190) and claudin-low (MDA-MB-231, Hs578T). All cell lines were cultured according to ATCC guidelines in a humidified incubator at 37°C and 5% CO_2_, except for MDA-MB-453, which required zero percent CO_2_.

### Immunomagnetic enrichment and fluorescence activated cell sorting (IE/FACS)

100,000 cells were spiked into PBS and PB from healthy female donors. IE/FACS was performed as previously described [20] but with an emphasis on the preservation of RNA to be isolated directly from CTC mimic cells. FACS sorting was performed using a FACS Aria II (BD Biosciences, San Jose, CA) and a gating strategy devised based on negative controls (*n* = 23) (Supplementary Figure S2). All specimens were analyzed using consistent gates (Supplementary Figure S2) and single antibody-fluorochrome compensation controls prepared for each experiment. Samples were processed immediately following blood draws and all lysates were immediately placed on ice. Absolute cell counts and recovery rates were determined using the TruCOUNT method (BD Biosciences) with acquisition of 35,000 beads. Positive cells, identified as EpCAM positive, thioflavin positive, and CD45 negative, were sorted into 1 ml RNA Protect Cell Reagent (Qiagen, Hilden, Germany). A threshold of a single cell meeting these criteria qualified as a positive test result. For subtype specific characterization of all 10 cell lines based on cell surface marker expression, single antibody-fluorochrome staining's were performed using HER2 (FITC), EpCAM (PE), CDH1 (PerCP-Cy5.5), EGFR (Alexa Fluor 647), CD45 (PE-Cy7) and Thioflavin (Pacific Orange). Unstained cells served as negative controls for each antibody. Using FlowJo data analysis software (Ashland, OR), MFI was calculated based on gating of stained versus unstained populations.

### RNA flow cytometry

RNA flow cytometry was performed following the previously published method by Hanley *et al*. [34]. This technology allows for the detection of single mRNA molecules of the transcripts of interest using simultaneous signal amplification of branched DNA and background suppression. Briefly, cells were fixed with 1% paraformaldehyde (Electron Microscopy Sciences, Hatfield, PA) for 10 min at room temperature (RT). Cell permeabilization was performed for 15 min at RT in BD FACS lysing solution (BD Biosciences) containing 0.2% saponin (Sigma, St. Louis, MO). Sequential hybridizations were performed in microcentrifuge tubes with target-specific probes, pre-amplifiers, amplifiers, and fluorophore conjugated labeled probes. The target probes were multiplexed and included HER2 (BV421), ER (APC), a mesenchymal cocktail containing FN1, CDH2, and SERPINE1 (FITC), and an epithelial cocktail containing CK8, CK14, CK17, CK18, CK19, CK20, EpCAM, and MUC1 (PE-YG). Flow cytometry data was acquired on a BD LSRFortessa (BD Biosciences, San Jose, CA). Probes and reagents were designed and manufactured by Advanced Cell Diagnostics (Hayward CA). Data were analyzed for the percentage of cells expressing the target probes as well as MFI.

### Helicos single-molecule RNA-seq

This technology enables nucleic acids quantitation and sequencing without ligation or amplification [35]. Genomic DNA is sheared, tailed with poly-A and hybridized to a flow-cell surface containing oligo-dT for initiating sequencing-by-synthesis. For BT474, total RNA was isolated using the Qiagen AllPrep Mini Kit (QIAGEN, Venlo, Netherlands). RNA from PB was isolated using the QIAamp RNA Blood Mini Kit (QIAGEN). Input RNA was directly hybridized to the flow cell allows for direct sequencing and quantitation of RNA molecules bound to the DNA probes. No PCR, sample selection or ligation is required, thus avoiding possible biases. Each tailed cDNA sample is injected into one of 50 flow-cell channels and sequenced on a Helicos Genetic Analysis System (HeliScope).

### Illumina hiSeq RNA-seq

For sample preparation from low cell number and BT474 small total RNA inputs (10ng-10pg) the Ovation Single Cell RNA-Seq System (NuGEN, San Carlos, CA) was used. For the BT474 bulk cells and PB cell total RNA isolation was performed using the AllPrep Mini Kit and QIAamp RNA Blood Mini Kit (QIAGEN) and library preparation for RNA-Seq was performed using the Ovation RNA amplification system V2 (NuGEN) and Ultra Low Library System V2 were used (NuGEN). Briefly, 10-500pg of total RNA was first subjected to first strand cDNA synthesis using oligo-dT plus selective priming that targets non-ribosomal RNA sequences in the transcriptome. Nucleotide analog and the original template RNA were degraded, leaving only single stranded antisense cDNA fragments (average size of 230 nucleotides). The fragments are primed using a random octamer with the forward adaptor attached to the 5′ end. Following end repair, the reverse adaptor was ligated to the free end of the now double stranded cDNA, which was enriched for coding and regulatory sequences. A dedicated read barcode design was used for sample identification. Final amplification PCR yielded the strand-specific cDNA libraries. These libraries were sequenced as 100bp paired end reads using an Illumina HiSeq 2000.

### qRT-PCR

For reverse transcription the qScript cDNA SuperMix (Quanta Biosciences, Gaithersburg, MD) was used according to the user guide. RNA was isolated from BT474 cells using TRIzol reagent (Life Technologies, Carlsbad, CA) and 1ug of total RNA was used per 20 μl reaction. The TaqMan Gene Expression Master Mix (Applied Biosystems) was used for real time qRT-PCR and gene expression quantification and validation. All TaqMan gene expression assays (Applied Biosystems) used in this study are listed in Supplementary Table S1. The genes were selected to validate cell purity as well as based on a list of differentially expressed genes generated from the RNA-Seq analysis comparing sorted and bulk cancer cells to PB. All samples were run as technical duplicates and biological triplicates. Gene expression was normalized against glyceraldehyde 3-phosphate dehydrogenase (GAPDH) and differential expression calculated using the ΔCt-method (2^−ΔΔCt^) [36].

### Statistical analysis

One-way ANOVA with a Kruskal-Wallace test was used to analyze for differences in recovery rates and marker MFI between intrinsic subtypes (Prism, GraphPad Software Inc., La Jolla, CA). For the Helicos experiments, the reads were aligned to the human reference genome (hg18) using the Burrows-Wheeler aligner (BWA) [37]. This method corresponds to a maximum edit distance of 2 within 32 nucleotide long seed regions. Mismatch, gap open and gap extension penalties were set to 3, 11 and 4 respectively. For the Illumina experiments, TopHat 1 [38], which uses Bowtie 1 [39], was used to align the reads to hg18, using one mismatch. Reads were counted on exons and normalized as Reads Per Kilobase per Million mapped reads (RPKM). The RPKM values were first adjusted by globally matching the count distribution at the 75^th^ percentile and then adjusting counts to have a uniform distribution across all samples. Differential expression was calculated with a significance of *p* < 0.05 after Benjamini and Hochberg correction using a null model constructed from 1% of transcripts showing the closet average level of observation to estimated experimental noise. All NGS data files have been deposited within NCBI GEO with the accession number pending.

## CONCLUSION

The current study demonstrated that our EpCAM based IE/FACS CTC isolation strategy can efficiently capture most breast cancer subtypes. We further show that we could successfully analyze captured CTC mimic cells using whole transcriptome RNA sequencing. Combined, this could provide a powerful, unbiased “liquid biopsy” tool for breast cancer research and diagnosis to assess in real time a patient's unique tumor biology and inform treatment decisions.

## SUPPLEMENTARY FIGURES AND TABLE



## References

[1] Ferlay J, Soerjomataram I, Ervik M, Dikshit R, Eser S, Mathers C, Rebelo M, Parkin DM, Forman D, Bray F (2013). GLOBOCAN 2012 v1.0, Cancer Incidence and Mortality Worldwide: IARC CancerBase No. 11 [Internet].

[2] Hayes DF, Smerage J (2008). Is there a role for circulating tumor cells in the management of breast cancer?. Clin Cancer Res.

[3] Kasimir-Bauer S (2009). Circulating tumor cells as markers for cancer risk assessment and treatment monitoring. Mol Diagn Ther.

[4] Pantel K, Alix-Panabieres C, Riethdorf S (2009). Cancer micrometastases. Nat Rev Clin Oncol.

[5] McInnes LM, Jacobson N, Redfern A, Dowling A, Thompson EW, Saunders CM (2015). Clinical implications of circulating tumor cells of breast cancer patients: role of epithelial-mesenchymal plasticity. Front Oncol.

[6] Cristofanilli M, Budd GT, Ellis MJ, Stopeck A, Matera J, Miller MC, Reuben JM, Doyle GV, Allard WJ, Terstappen LW, Hayes DF (2004). Circulating tumor cells, disease progression, and survival in metastatic breast cancer. N Engl J Med.

[7] Allard WJ, Matera J, Miller MC, Repollet M, Connelly MC, Rao C, Tibbe AG, Uhr JW, Terstappen LW (2004). Tumor cells circulate in the peripheral blood of all major carcinomas but not in healthy subjects or patients with nonmalignant diseases. Clin Cancer Res.

[8] Zhang L, Riethdorf S, Wu G, Wang T, Yang K, Peng G, Liu J, Pantel K (2012). Meta-analysis of the prognostic value of circulating tumor cells in breast cancer. Clin Cancer Res.

[9] Lucci A, Hall CS, Lodhi AK, Bhattacharyya A, Anderson AE, Xiao L, Bedrosian I, Kuerer HM, Krishnamurthy S (2012). Circulating tumour cells in non-metastatic breast cancer: a prospective study. Lancet Oncol.

[10] Yu M, Bardia A, Wittner BS, Stott SL, Smas ME, Ting DT, Isakoff SJ, Ciciliano JC, Wells MN, Shah AM, Concannon KF, Donaldson MC, Sequist LV, Brachtel E, Sgroi D, Baselga J (2013). Circulating breast tumor cells exhibit dynamic changes in epithelial and mesenchymal composition. Science.

[11] Mayer JA, Pham T, Wong KL, Scoggin J, Sales EV, Clarin T, Pircher TJ, Mikolajczyk SD, Cotter PD, Bischoff FZ (2011). FISH-based determination of HER2 status in circulating tumor cells isolated with the microfluidic CEE platform. Cancer Genet.

[12] Coumans FA, van Dalum G, Beck M, Terstappen LW (2013). Filter characteristics influencing circulating tumor cell enrichment from whole blood. PLoS One.

[13] Parker JS, Mullins M, Cheang MC, Leung S, Voduc D, Vickery T, Davies S, Fauron C, He X, Hu Z, Quackenbush JF, Stijleman IJ, Palazzo J, Marron JS, Nobel AB, Mardis E (2009). Supervised risk predictor of breast cancer based on intrinsic subtypes. J Clin Oncol.

[14] Sorlie T, Tibshirani R, Parker J, Hastie T, Marron JS, Nobel A, Deng S, Johnsen H, Pesich R, Geisler S, Demeter J, Perou CM, Lonning PE, Brown PO, Borresen-Dale AL, Botstein D (2003). Repeated observation of breast tumor subtypes in independent gene expression data sets. Proc Natl Acad Sci U S A.

[15] Harrell JC, Prat A, Parker JS, Fan C, He X, Carey L, Anders C, Ewend M, Perou CM (2012). Genomic analysis identifies unique signatures predictive of brain, lung, and liver relapse. Breast cancer research and treatment.

[16] Sieuwerts AM, Kraan J, Bolt J, van der Spoel P, Elstrodt F, Schutte M, Martens JW, Gratama JW, Sleijfer S, Foekens JA (2009). Anti-epithelial cell adhesion molecule antibodies and the detection of circulating normal-like breast tumor cells. J Natl Cancer Inst.

[17] Prat A, Parker JS, Karginova O, Fan C, Livasy C, Herschkowitz JI, He X, Perou CM (2010). Phenotypic and molecular characterization of the claudin-low intrinsic subtype of breast cancer. Breast Cancer Res.

[18] Creighton CJ, Li X, Landis M, Dixon JM, Neumeister VM, Sjolund A, Rimm DL, Wong H, Rodriguez A, Herschkowitz JI, Fan C, Zhang X, He X, Pavlick A, Gutierrez MC, Renshaw L (2009). Residual breast cancers after conventional therapy display mesenchymal as well as tumor-initiating features. Proc Natl Acad Sci U S A.

[19] Magbanua MJ, Sosa EV, Roy R, Eisenbud LE, Scott JH, Olshen A, Pinkel D, Rugo HS, Park JW (2013). Genomic profiling of isolated circulating tumor cells from metastatic breast cancer patients. Cancer Res.

[20] Magbanua MJ, Park JW (2013). Isolation of circulating tumor cells by immunomagnetic enrichment and fluorescence-activated cell sorting (IE/FACS) for molecular profiling. Methods.

[21] Gonzalez-Angulo AM, Ferrer-Lozano J, Stemke-Hale K, Sahin A, Liu S, Barrera JA, Burgues O, Lluch AM, Chen H, Hortobagyi GN, Mills GB, Meric-Bernstam F PI3K pathway mutations and PTEN levels in primary and metastatic breast cancer. Mol Cancer Ther.

[22] Gutierrez MC, Detre S, Johnston S, Mohsin SK, Shou J, Allred DC, Schiff R, Osborne CK, Dowsett M (2005). Molecular changes in tamoxifen-resistant breast cancer: relationship between estrogen receptor, HER-2, and p38 mitogen-activated protein kinase. J Clin Oncol.

[23] Mobbs BG, Fish EB, Pritchard KI, Oldfield G, Hanna WH (1987). Estrogen and progesterone receptor content of primary and secondary breast carcinoma: influence of time and treatment. Eur J Cancer Clin Oncol.

[24] Idirisinghe PK, Thike AA, Cheok PY, Tse GM, Lui PC, Fook-Chong S, Wong NS, Tan PH (2010). Hormone receptor and c-ERBB2 status in distant metastatic and locally recurrent breast cancer. Pathologic correlations and clinical significance. Am J Clin Pathol.

[25] Niikura N, Liu J, Hayashi N, Mittendorf EA, Gong Y, Palla SL, Tokuda Y, Gonzalez-Angulo AM, Hortobagyi GN, Ueno NT (2012). Loss of human epidermal growth factor receptor 2 (HER2) expression in metastatic sites of HER2-overexpressing primary breast tumors. J Clin Oncol.

[26] Meng S, Tripathy D, Shete S, Ashfaq R, Haley B, Perkins S, Beitsch P, Khan A, Euhus D, Osborne C, Frenkel E, Hoover S, Leitch M, Clifford E, Vitetta E, Morrison L (2004). HER-2 gene amplification can be acquired as breast cancer progresses. Proc Natl Acad Sci U S A.

[27] Lang JE, Scott JH, Wolf DM, Novak P, Punj V, Magbanua MJ, Zhu W, Mineyev N, Haqq CM, Crothers JR, Esserman LJ, Tripathy D, van't Veer L, Park JW (2014). Expression profiling of circulating tumor cells in metastatic breast cancer. Breast cancer research and treatment.

[28] Marrinucci D, Bethel K, Bruce RH, Curry DN, Hsieh B, Humphrey M, Krivacic RT, Kroener J, Kroener L, Ladanyi A, Lazarus NH, Nieva J, Kuhn P (2007). Case study of the morphologic variation of circulating tumor cells. Hum Pathol.

[29] Barriere G, Tartary M, Rigaud M (2012). Epithelial mesenchymal transition: a new insight into the detection of circulating tumor cells. ISRN Oncol.

[30] Scheel C, Weinberg RA (2012). Cancer stem cells and epithelial-mesenchymal transition: concepts and molecular links. Semin Cancer Biol.

[31] Ansieau S (2013). EMT in breast cancer stem cell generation. Cancer Lett.

[32] Lang JE, Wecsler JS, Press MF, Tripathy D (2014). Molecular markers for breast cancer diagnosis, prognosis and targeted therapy. J Surg Oncol.

[33] Holliday DL, Speirs V (2011). Choosing the right cell line for breast cancer research. Breast Cancer Res.

[34] Hanley MB, Lomas W, Mittar D, Maino V, Park E (2013). Detection of low abundance RNA molecules in individual cells by flow cytometry. PLoS One.

[35] Thompson JF, Steinmann KE Single molecule sequencing with a HeliScope genetic analysis system. Curr Protoc Mol Biol.

[36] Livak KJ, Schmittgen TD (2001). Analysis of relative gene expression data using real-time quantitative PCR and the 2(-Delta Delta C(T)) Method. Methods.

[37] Li H, Durbin R (2009). Fast and accurate short read alignment with Burrows-Wheeler transform. Bioinformatics.

[38] Trapnell C, Pachter L, Salzberg SL (2009). TopHat: discovering splice junctions with RNA-Seq. Bioinformatics.

[39] Langmead B, Trapnell C, Pop M, Salzberg SL (2009). Ultrafast and memory-efficient alignment of short DNA sequences to the human genome. Genome Biol.

