# Supplementary material for: EpCAM based capture detects and recovers circulating tumor cells from all subtypes of breast cancer except claudin-low
Source: Oncotarget. 2015 Oct 19;6(42):44623–34. doi: 10.18632/oncotarget.5977 (PMC4792580; doi:10.18632/oncotarget.5977)
Supplement: Supplementary file 1 [file oncotarget-06-44623-s001.pdf]

SUPPLEMENTARY FIGURES AND TABLE

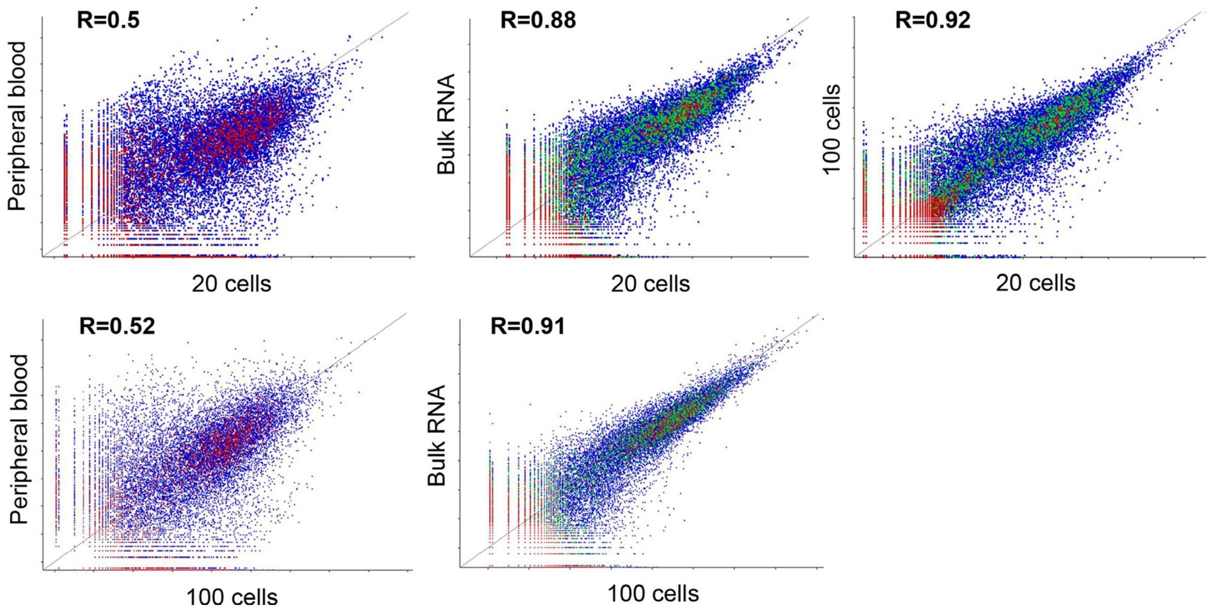

Supplementary Figure S1: Illumina high seq. data. Comparing low (20 cells) and high (100 cells) sorted BT474 cells to each other and to bulk BT474 RNA demonstrated high correlation (20 cells vs. bulk. $R = 0.88$; 100 cells vs. bulk, $R = 0.91$; 20 cells vs. 100 cells, $R = 0.92$). Comparison to PB RNA showed poor correlation (PB vs. 20 cells, $R = 0.5$; PB vs. 100 cells, $R = 0.52$) (Pearson's correlation analysis; both x and y-axis represent RPKM ($\log_2$) values).

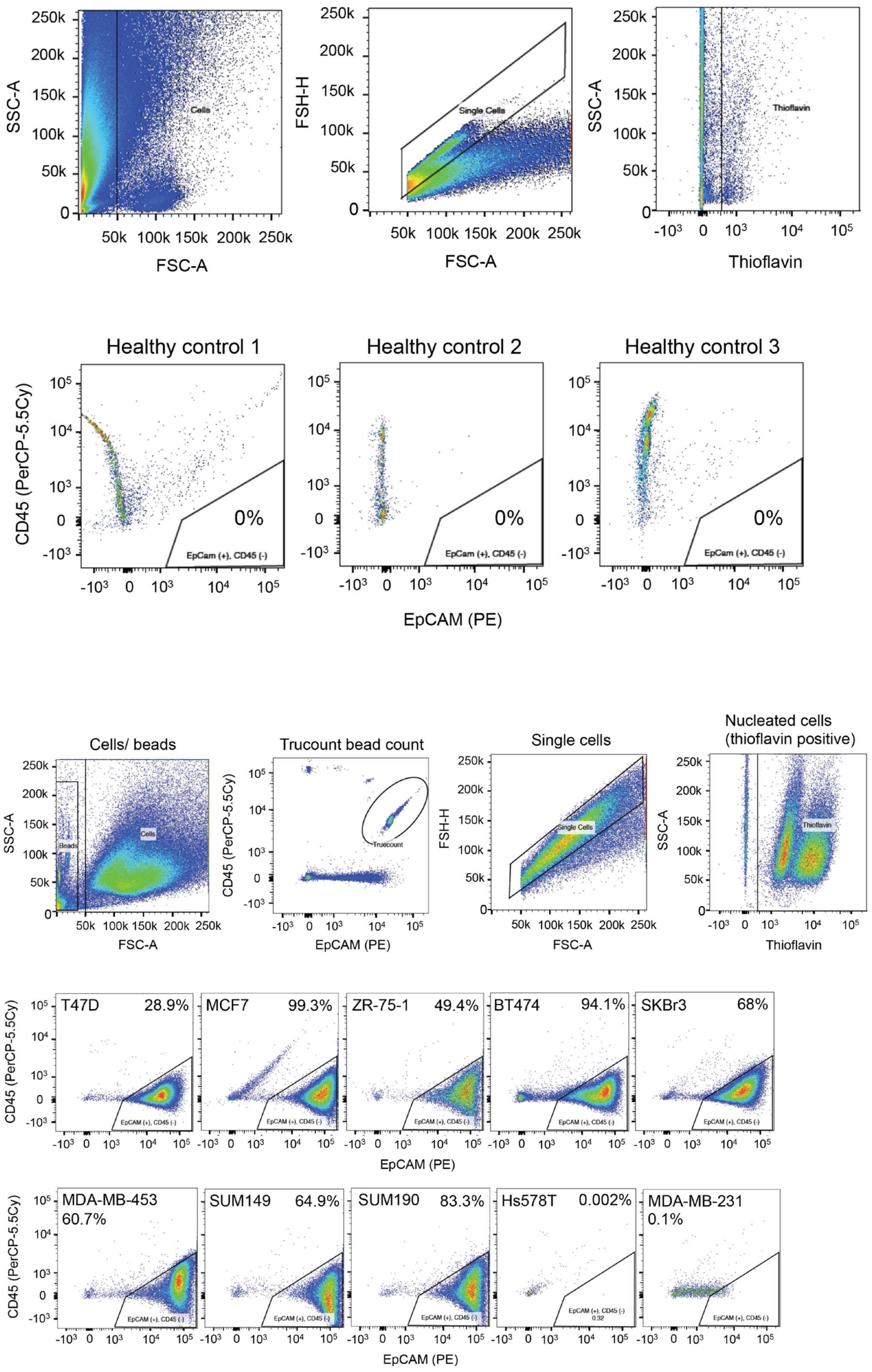

Supplementary Figure S2: Devising a FACS gating strategy to exclude blood cells and maximize EpCAM-positive cell recovery. A. IE/FACS data from three representative healthy donor blood samples (EpCAM positive, CD45 negative cells 0%, n = 3). **B.** IE/FACS data for recovery rates from PBS for all ten cell lines. (*Continued*)

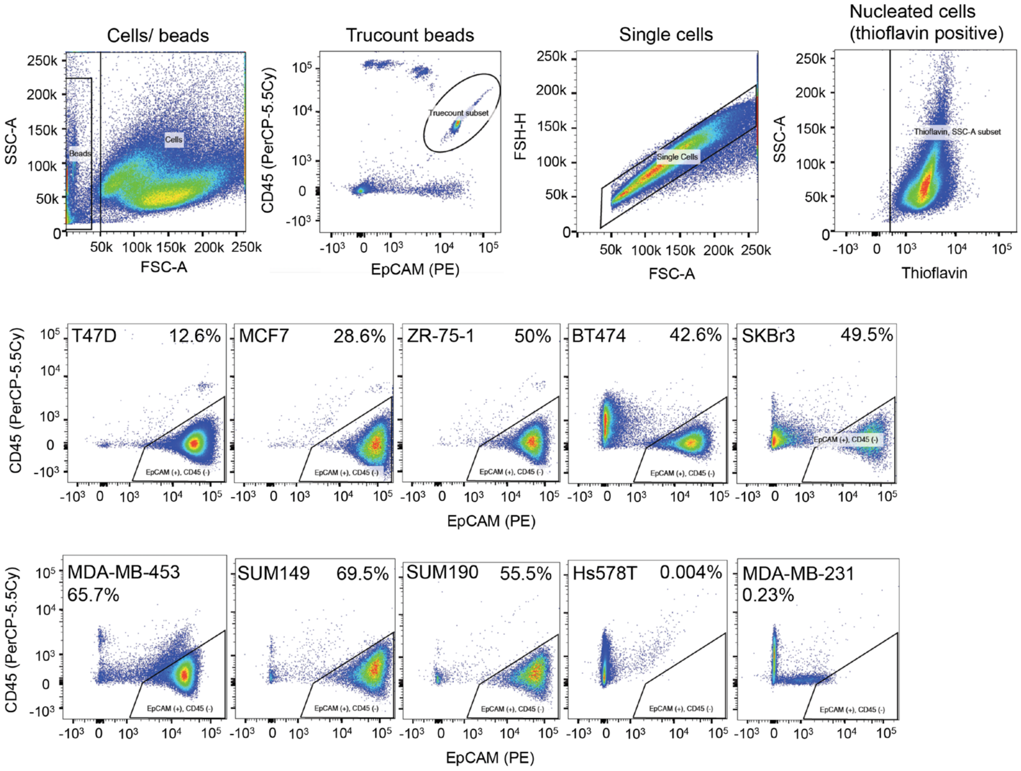

Supplementary Figure S2: (*Continued*) Devising a FACS gating strategy to exclude blood cells and maximize EpCAM-positive cell recovery. C. IE/FACS data for recovery rates from peripheral blood for all ten cell lines.

Supplementary Table S1: TaqMan gene expression assays

Gene	Assay number	Amplicon length
EGFR	Hs01076078_m1	60
PGR	Hs01556702_m1	77
GREB1	Hs005536409_m1	77
ESRP1	Hs00214472_m1	114
SYBU/GOLSYN	Hs01052028_m1	81
MAL2	Hs00294541_m1	63
TOM1L1	Hs01098794_m1	65
KRT8	Hs01595539_m1	164
PRLR	Hs01061477_m1	107
AGR2	Hs00356521_m1	69
AIF1	Hs00610419_g1	92
CASP1	Hs00354836_m1	76
PTPRC/CD45	Hs04189704_m1	57
PECAM1/CD31	Hs00169777_m1	65
FCN1	Hs00157572_m1	105
EPCAM	Hs00901885_m1	95
GAPDH	Hs02758991_g1	93

All primers span exon junctions.
